# Supplementary material for: Freestanding non-covalent thin films of the propeller-shaped polycyclic aromatic hydrocarbon decacyclene
Source: Nat Commun. 2022 Apr 8;13:1920. doi: 10.1038/s41467-022-29429-8 (PMC8993932; doi:10.1038/s41467-022-29429-8)
Supplement: Supplementary file 1 — Supplementary Information [file 41467_2022_29429_MOESM1_ESM.pdf]

Supplementary information for Freestanding Non-covalent Thin Films of the Propeller-shaped Polycyclic Aromatic Hydrocarbon Decacyclene

Alex van der Ham[1,4], Xue Liu[1,2,4], Dario Calvani[1], Adéla Melcrová[3], Melania Kozdra[1], Francesco Buda[1], Herman S. Overkleeft[1], Wouter H. Roos[3], Dmitri V. Filippov[1], and Grégory F. Schneider[1,*]

1 Leiden Institute of Chemistry, Leiden University, Einsteinweg 55, 2333 CC Leiden (The Netherlands)
* e-mail: g.f.schneider@chem.leidenuniv.nl

2 State Key Laboratory for Mechanical Behavior of Materials, Xi'an Jiaotong University, 710049 Xi'an, China.

3 Zernike Institute for Advanced Materials, Rijksuniversiteit Groningen, Nijenborgh 4, 9747 AG Groningen (The Netherlands)

4 These authors contributed equally to this work

Experimental

Sample preparation 3

Thin film characterization 5

AFM indentation experiments 6

DFT computations 9

Molecular Dynamics 9

Figure S1: 1H NMR spectra of decacyclene 4

Figure S2: UV-Vis spectrum of decacyclene 4

Figure S3: Infrared spectra of decacyclene 4

Figure S4: TEM and electron diffraction images 6

Figure S5: Additional AFM images of the decacyclene films 6

Figure S6: AFM images before and after nanoindentation 7

Figure S7: Height profile of the decacyclene film spanning over an aperture 7

Figure S8: Representative force-indentation curves 7

Figure S9: Mechanical parameters from the AFM nanoindentation experiments 8

Figure S10: Potential energy landscape for the interconversion of decacyclene 9

Figure S11: Validation of the MD water box 11

Figure S12: Fluctuations of the MD simulation box 11

Figure S13: Comparison of computed isotherms for racemate *versus* enantiomer pure 12

Table S1: OPLS atom types for decacyclene 11

DFT Geometries 13

Supplementary references 20

1. Supplementary Notes

Decacyclene was synthesized and purified according to the procedure of Amick and Scott.[1, 2] Identity and purity of the material were checked using ^{1}H NMR spectroscopy on a Bruker DPX 300 NMR instrument equipped with a BBFO probe head for 5 mm outer diameter tubes. Spectra were recorded at 300 MHz for ^{1}H using tetrachloroethane-d_2 obtained from a commercial source (SigmaAldrich) which was distilled before use. Solubility was found too low to allow recording of a ^{13}C spectrum. NMR spectra were processed using the MestrReNova© 14.1.0 software suite. FTIR spectra were recorded on a Perkin-Elmer Paragon 1000 FTIR spectrophotometer equipped with a Golden Gate attenuated total reflection (ATR) device. All spectra were found to be in accordance with literature. Melting points were recorded on a Stuart scientific SMP3 melting point apparatus and are uncorrected. Thin film samples were prepared on a Langmuir–Blodgett trough (KSV NIMA, Finland) filled with Millipore® Ultrapure water (18 MΩ cm^{-1}). In a typical experiment, decacyclene was dissolved in chloroform and filtered over a syringe filter (0.45 µm, PTFE, VWR™) to yield a clear brown solution. Concentrations were back-calculated from evaporated aliquots. Typical concentrations were on the order of 1 mM. A small amount of this solution (75 µL) was carefully spread at the air:water interface using a micropipette. After 30 min, the surface was compressed by two barriers with a fixed speed of 2 mm min^{-1} to reach a certain surface. The surface pressure was measured with a Wilhelmy balance. Thin film samples were transferred onto silicon wafers at constant pressure by the Langmuir–Schäfer method. Samples were transferred onto copper or gold TEM grids following standard procedures.[3] Copper foil was obtained from Puratonic® and had a thickness of 0.025 mm and a 99.999% purity.

2. Supplementary Methods

2.1.1 Synthetic procedures

Decacyclene. A solution of acenaphthone (250 mg, 1.5 mmol), *p*-TsOH (1 g, 5.26 mmol, 3.5 eq.) and propionic acid (0.4 ml, 5.4 mmol, 3.6 eq.) in 1,2-dichlorobenzene (1.5 ml) was stirred at 105°C for 16 hrs. The reaction mixture was then poured into methanol, and neutralized by the dropwise addition of 5 M NaOH. The precipitate was collected by filtration, washed with MeOH and heptane, and then allowed to dry in contact with air to provide the title compound as a light brown powder. Yield: 157 mg, 0.35 mmol, 23.3%. ^{1}H NMR (500MHz, $C_2D_2Cl_4$) δ 8.78 (d, J = 9.0 Hz, 6H, H-3), 7.99 (d, J = 9.0 Hz, 6H, H-1), 7.83 (t, J = 9.0 Hz, 6H, H-2). HRMS (ESI-TOF) m/z: calc'd for $C_{36}H_{19}$ $[M+H]^+$: 451.14868 found 451.14813. m.p. > 300 °C. For NMR, UVVis and IR spectra see Fig. S1 – S3, respectively.

2.1.2 Compound characterization

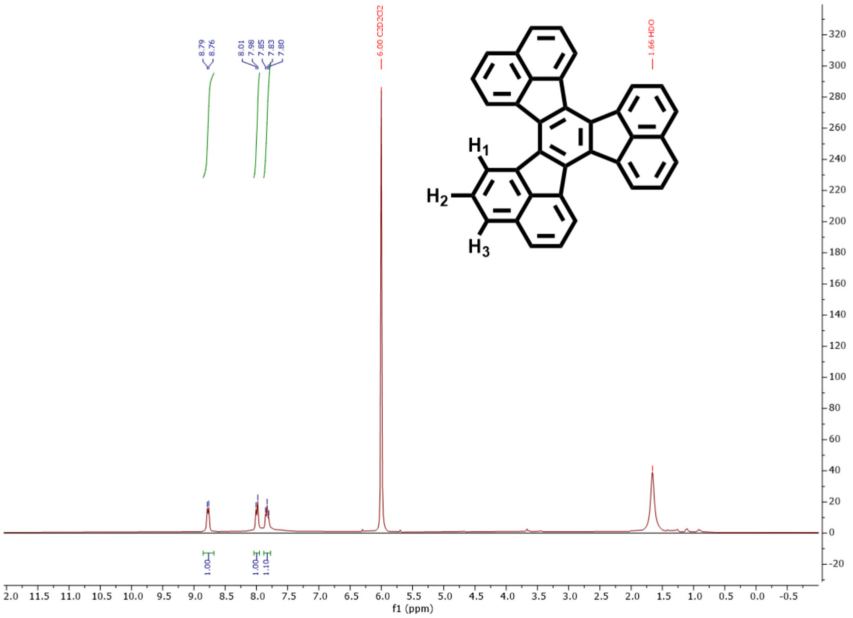

Supplementary Figure 1. ^{1}H NMR spectrum of decacyclene in $C_2D_2Cl_4$.

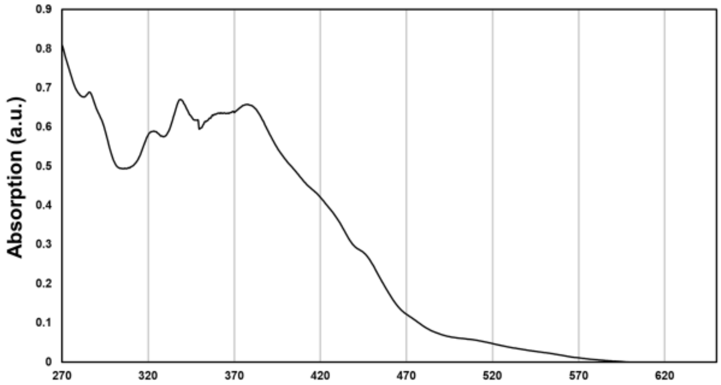

Supplementary Figure 2. UV-Vis spectrum of decacyclene in $CHCl_3$.

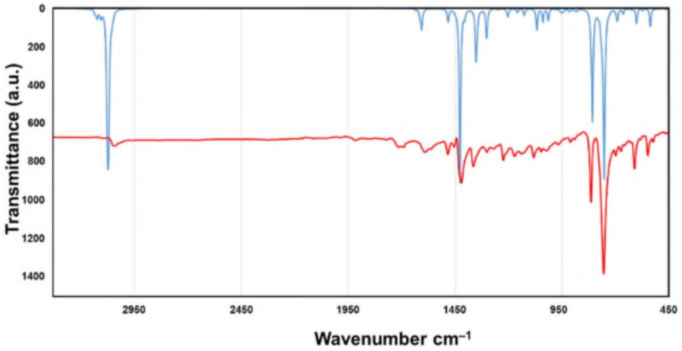

Supplementary Figure 3. Infrared spectra of decacyclene as computed for the dimer (Structure S5) at the PBE/6-31G(d,p) level (blue), and experimentally recorded for the powder (red).

2.1.3 Thin film characterization

SEM images were recorded by using an FEI NOVA nano SEM 200 scanning electron microscope. Samples intended for indentation experiments were only imaged on a small part of the TEM grids, to prevent contamination by electron beam exposure. TEM experiments were conducted on an image-side Cs-corrected FEI Titan 80–300 microscope operated at 300 kV (Fig. S4). AFM images of the thin film on the Si/SiO_2 wafer were recorded on a JPK Nano Wizard Ultra Speed machine with a silicon 254 probe (AC 160 TS, Asylum Research) with 300 kHz nominal resonance frequency (Fig. S5). The images were scanned in intermittent contact mode in air at room temperature. Both the AFM images and the force curves were processed using JPK Data Processing software.

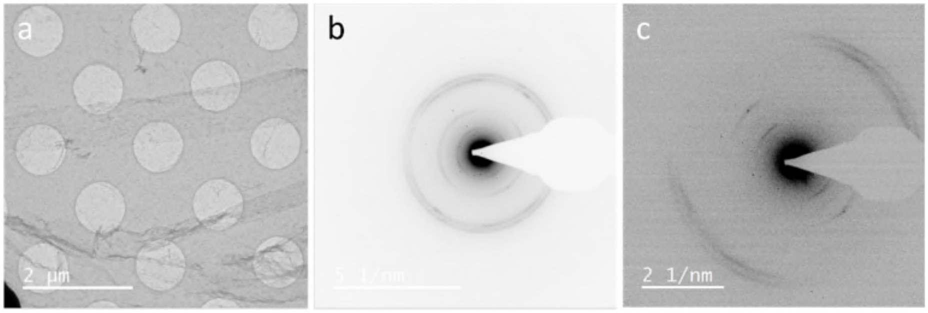

Supplementary Figure 4. **a**, Representative bright-field TEM and **b**, and **c**, electron-diffraction patterns obtained from decacyclene films, showing absence of global crystallinity. Beam energy 300.0 kV, probe current 8.31 nA.

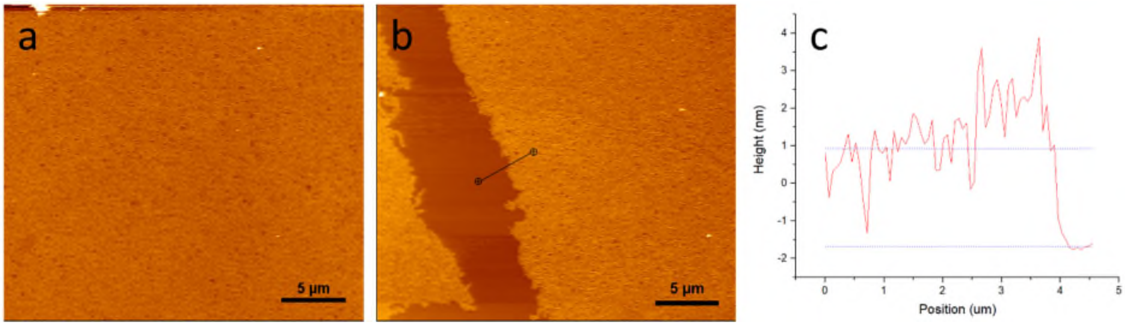

Supplementary Figure 5. AFM characterization of decacyclene films. **a**, and **b**, representative AFM images of separately prepared decacyclene film samples, showing their homogeneity. **c**, height profile of the sample shown in **b**.

2.1.4 AFM nanoindentation experiments

AFM imaging and nanoindentation of the TEM grid supported freestanding thin films was performed with an JPK Nano Wizard Ultra Speed AFM.[4, 5] The TEM grid (with apertures of 1 μm in diameter) was immobilized by tape on top of a glass slide and imaged in QI™ mode. The experiments were performed in air at room temperature (22°C) using SNL-10A cantilevers (Bruker) with a calibrated spring constant of 0.432 ± 0.003 Nm^{-1} and a silicon nitride tip with nominal radius of 2 nm. The imaging force was ~400–600 pN. After imaging the free-standing thin films, the intact films were indented in the center by a force up to 50 nN with an indentation velocity of 300 nms^{-1}. Force-distance curves were recorded during indentation. After indentation, the same spot was imaged again. Representative images before and after nanoindentation are shown at Fig. S6. The height profile of a thin film spanning over a circular aperture is plotted in Fig. S7. The profile exhibits a dip in height in the middle, as also previously reported for suspended graphene membranes,[4] and a small elevation of the film height around the circumference of the aperture.

2.1.5 AFM force-indentation curves analysis

In brief, the nanoindentation experiment yields the deflection of the cantilever as a function of the Z-piezo displacement. This dependency was converted to the applied force versus the vertical distance between the tip and the sample surface by subtracting the deflection of the cantilever itself.[6] The contact point between the tip and the film was defined as a point, when the force-indentation curve starts exhibiting non-zero force. The initial increase in the force-indentation response up to the first apparent change in the slope or a small drop in the force (< 2 nN) was fitted using:

$$\sigma = E\varepsilon + D\varepsilon^2 \qquad \text{(Eq. S1)}$$

according to the model for elastic properties of a 2D material as described in Ref [4]. Two parameters were derived from the fit: prestress in 2D and elastic modulus in 2D. By dividing the 2D elastic modulus by the film thickness h = 2.5 nm, we obtained the effective Young's modulus of the decacyclene film. The analysis was done independently on two sets of data, the curves with the small drop in the force increase (Fig. S8a; N = 7), and the curves with apparent change in the slope (Fig. S8b; N = 20). Fig. S9 shows the histograms of the pretension in 2D, the effective Young's modulus, the force inducing non-elastic changes in the film (when a small drop or slope change occur), and the force needed to fully rupture the thin film. The latter is being defined as a force drop > 2 nN. The histograms show comparable distributions for the two subsets of data and hence the data from both subsets were combined and included into overall statistical analysis of the mechanical properties of the decacyclene films.

2.1.6 AFM indentation experiments

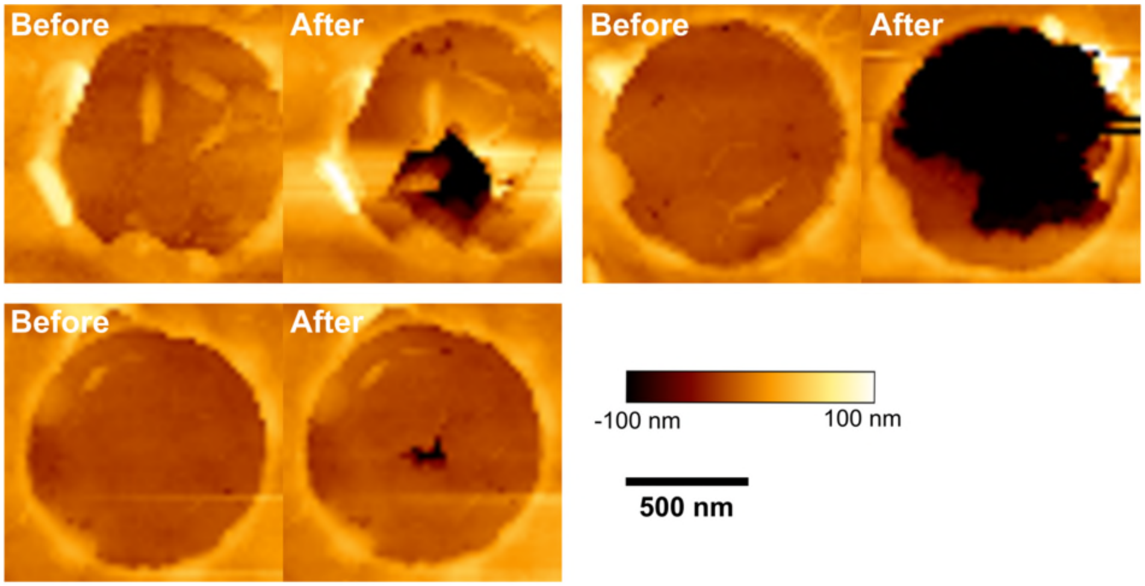

Supplementary Figure 6. Representative AFM images of the decacyclene film spanning over the circular apertures before and after the nanoindentation with a force load of 50 nN.

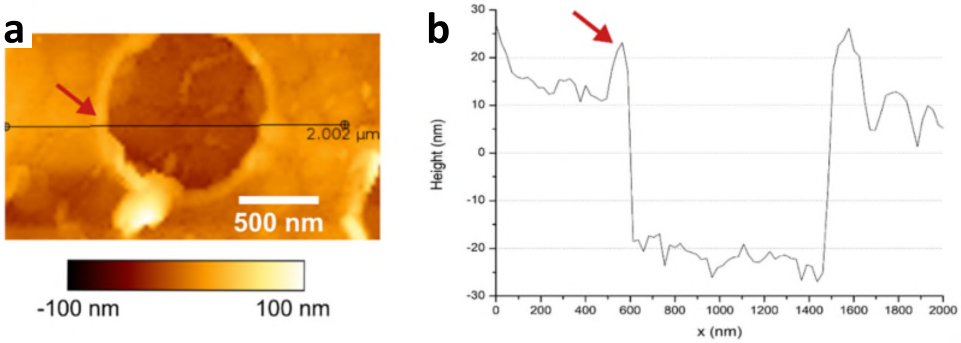

Supplementary Figure 7. a, AFM image of a thin film spanning over the circular aperture with a cross-section line highlighted. **b**, corresponding height profile along the cross-section line. Red arrows in both panels point to the increase in height at the rim of the aperture, visible as a lighter color in the AFM image.

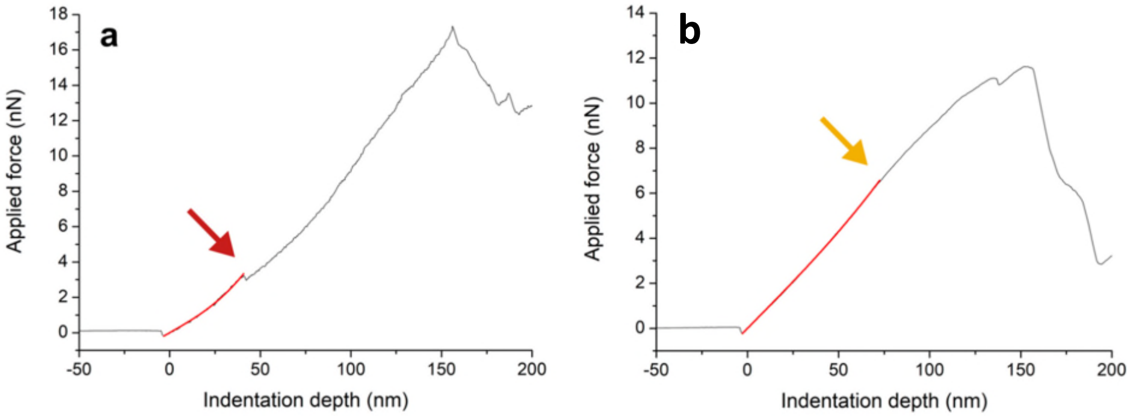

Supplementary Figure 8. Representative force-indentation curves. **a**, Curves displaying a small drop in the applied force (red arrow), and **b**, change in the slope (yellow arrow). Red lines represent fits of the experimental data according to Eq. S3.[4]

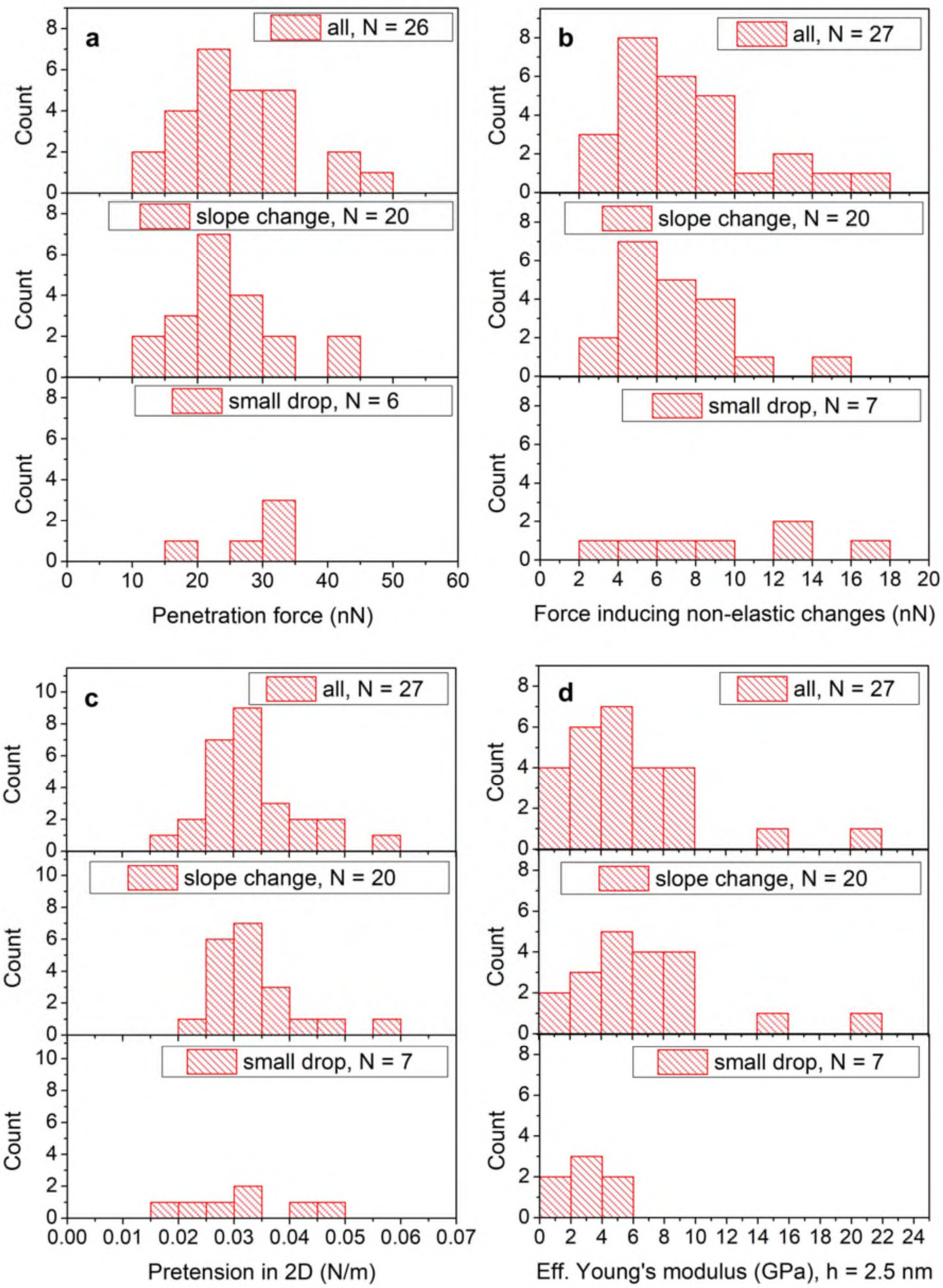

Supplementary Figure 9. Mechanical parameters from the AFM nanoindentation experiments. Histograms of the parameters from all the acquired force-indentation curves, from the subset of the curves with an apparent change in the slope, and from the subset with the small drop in the force are shown. **a**, Penetration force needed to fully rupture the thin film (demonstrated as a drop in the applied force > 2 nN). **b**, Force inducing non-elastic changes. *i.e.* the force when the first small drop or the slope change occurs. **c**, Pretension in 2D material as defined in Ref. [4]. **d**, Effective Young's modulus of decacyclene film.

2.2 **Density Functional Theory**

2.2.1 Computational Methods

Equilibrium geometries were computed at the PBE/6-31G(d,p) level of theory using the Gaussian 09 Rev. D.01 program suite,[7] using the D3(BJ) dispersion correction.[8, 9] The geometry convergence criteria were set to tight (Opt = tight; Max. Force = $1.5 \cdot 10^{-7}$, Max. Displacement = $6.0 \cdot 10^{-7}$), and an internally defined super-fine grid size was used (SCF=tight, Int=VeryFineGrid), which is a pruned 175,974 grid for first-row atoms and a 250,974 grid for all other atoms. Free Gibbs energies were computed using Equation S2, in which ΔE_{gas} is the gas-phase energy (electronic energy) and $\Delta G^{T}_{\text{gas,QH}}$ (T = 293.15 K, p = 1 atm., C = 1 M) is the sum of corrections from the electronic energy to the free Gibbs energy in the quasi-harmonic oscillator approximation, including zero-point-vibrational energy. The $\Delta G^{T}_{\text{gas,QH}}$ were computed using the quasi-harmonic approximation in the gas phase according to the work of Truhlar in which vibrational frequencies lower than 100 cm^{-1} were raised to 100 cm^{-1} to correct for the breakdown of the harmonic oscillator model for the free energies of low-frequency vibrational modes.[10, 11] Stationary points were checked to have no imaginary frequencies for local minima, and one imaginary frequency for the transition state structure. All DFT structures were illustrated using CYLview.[12]

$$\Delta G^{T}_{\text{gas}} = \Delta E_{\text{gas}} + \Delta G^{T}_{\text{gas,QH}} \qquad \text{(Eq. S2)}$$

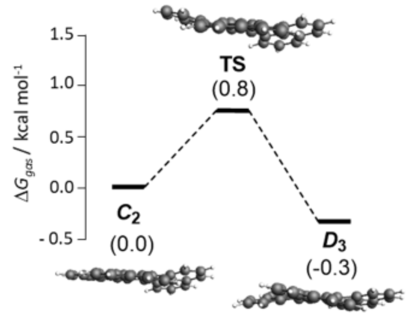

Supplementary Figure 10. Potential energy surface for the interconversion of decacyclene between its C_2 and D_3 conformer. Energies are expressed as gas-phase Gibbs free energies in kcal mol^{-1}, as computed at PBE-D3(BJ)/6-31G(d,p).

2.2.2 Molecular Dynamics

All MD simulations were carried out using GROMACS 2016 software suite.[13, 14, 15, 16, 17, 18, 19] The Particle Mesh Ewald (PME) method was employed to accurately account for electrostatic interactions.[20] The cut-off for Coulomb and Lennard-Jones interactions was set to 10 Å. During the NVT simulation the temperature was kept fixed with the V-rescale coupling method.[21]

The model for a body of water, comprised of 4139 water molecules, was simulated in a periodic box (5.0 x 5.0 x 20.0 nm^3) using the TIP4P-Ew/2004 force field.[22] The water surface tension was used as defined within the GROMACS software suite (Equation S3):

$$\gamma_{water} = \frac{1}{2} L_z [P_{zz} - \frac{1}{2}(P_{xx} + P_{yy})] \qquad \text{(Eq. S3)}$$

where L_z is the box length in the z direction, P_{xx}, P_{yy} and P_{zz} are the respective xx, yy and zz element of the pressure tensor,[23] and the ½ originates from the presence of two x-y plane surfaces in the system. The system was first energetically minimized and then equilibrated for 8 ns with NVT at 300 K to obtain

an average surface tension of γ_{water} = 58.46 mN m^{-1}. This value is in good agreement with previous studies.[24, 25] The water model was further validated using a radial distribution function and density analysis (SI Fig. S11).
Decacyclene molecules were simulated using the OPLS-AA force field (see Table S1)[26, 27, 28] and LigParGen[29] parameterization with charges calculated with the CM5 model[30] using the Gaussian 16 Rev. C.01 program suite.[31] Input geometries for decacyclene were obtained from DFT as described above.

2.2.3 Simulation run
Random input positions of 30 or 60 decacyclene molecules on both water surfaces were generated using the PACKMOL18 program.[32, 33] Having two independent water-decacyclene interfaces provides a more symmetric MD simulation box and increases the statistics of the results by averaging on both interfaces. Moreover, the presence of the decacyclene molecule on both sides avoids the diffusion of the water molecule through the periodic boundary condition along the z-axis. The two interfaces can be considered independent due to the thickness (5 nm) of the water box and the vacuum space (at least 5 nm on each side) above each interfaces along the z-axis. For each simulation, the system was first equilibrated with NVT at 70 K for 5 ns, and the temperature then raised to 300 K for another 5 ns. After these pre-equilibration simulations, the production run consists in NVT simulations were ran at 300 K for 10 ns. The final configuration extracted from the production run was used as a starting point for the subsequent NPT surface tension simulations. These series of simulations are repeated independently three times starting from a different random packing, in order to check the effect of initial conditions on the final results.

2.2.4 Surface Tension calculations
Surface tension coupling for surfaces parallel to the xy-plane was used. Uses normal pressure coupling for the z-direction, while the surface tension is coupled to the x/y dimensions of the box. The surface pressure was then increased stepwise, to generate the pressure-area isotherm. At each chosen surface pressure (0, 3, 10, 20, 30, 40, 50 mN m^{-1}), the surface tension coupling molecular dynamics has been performed while monitoring the change of the area in the x-y plane. In order to execute the constant surface tension simulations, the Berendsen pressure coupling was used.[34] For this coupling methods to be effective, a value for the compressibility is required, which is close to the real compressibility of the system, namely 4.5 x 10^{-5} bar^{-1}.[35] Simulations were stopped after 10 ns at which point an equilibrium state was reached. Data presented in the manuscript was obtained averaging the values on the last 10 ns of each corresponding simulation. The x-y area values are equilibrated enough with an error around ±0.05 Å. (Fig. S12). These series of simulations were repeated independently starting from each last equilibration simulation described in the previous paragraph, in order to increase the reproducibility of our results.

To verify whether a racemic mixture of decacyclene molecules gives the same isotherm as that of a homochiral system consisting of 60 (+) molecules, a system was modelled containing, above and below the water box, a mixture of 30 (+) rotating and 30 (–) molecules (Structures S1 and S2). No significant differences between the two systems was found (Fig. S13).

All MD results were illustrated using Visual Molecular Dynamics (VMD).[36]

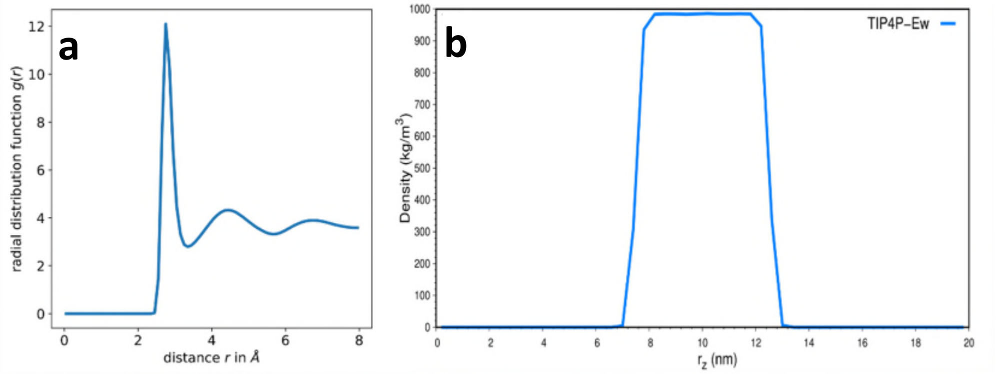

Supplementary Figure 11. **a**, O-O radial distribution function $g(\mathrm{r})$ for water, calculated using the MDAnalysis tool.[37] **b**, Density curve (kg m^{-3}) of the water molecules along the z-coordinate r_z (nm) of the water box.

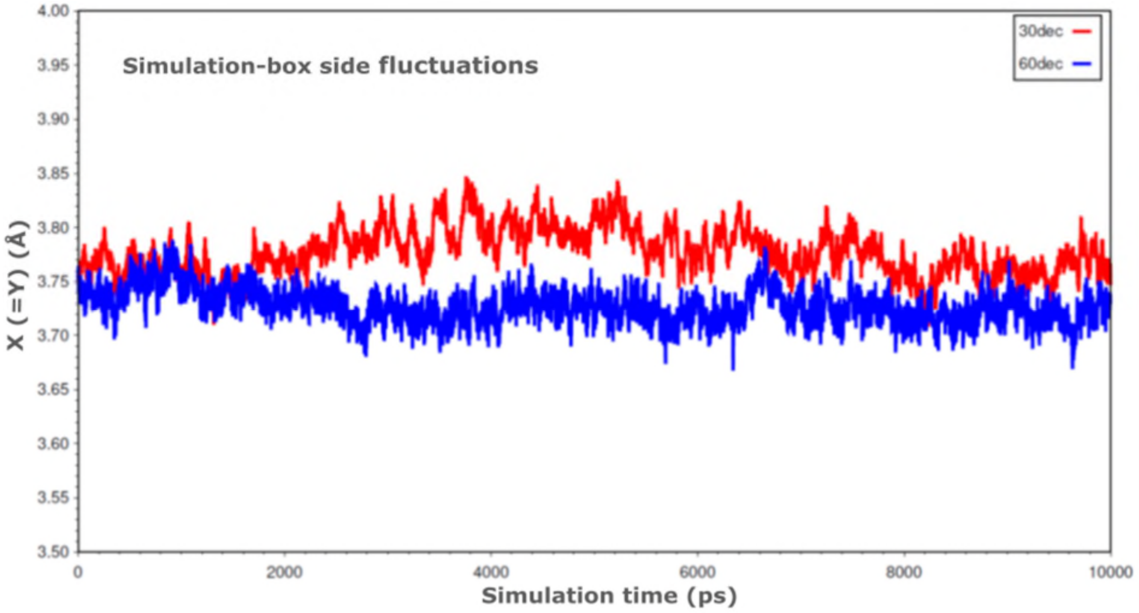

Supplementary Figure 12. Fluctuations of the simulation box side X (= Y) during the Surface Tension MD at 30 mN m^{-1} for 30 and 60 decacyclene systems, respectively in red and blue color. The simulation time-is reported in ps.

Supplementary Table 1. Assigned force field atom types for decacyclene molecule list for reproduce the simulation.[38]

**OPLS-AA atom type**	**Description**
opls_145	Benzene C - 12 site.[39] Use #145B for biphenyl
opls_146	Benzene H - 12 site.
opls_145B	Biphenyl C1

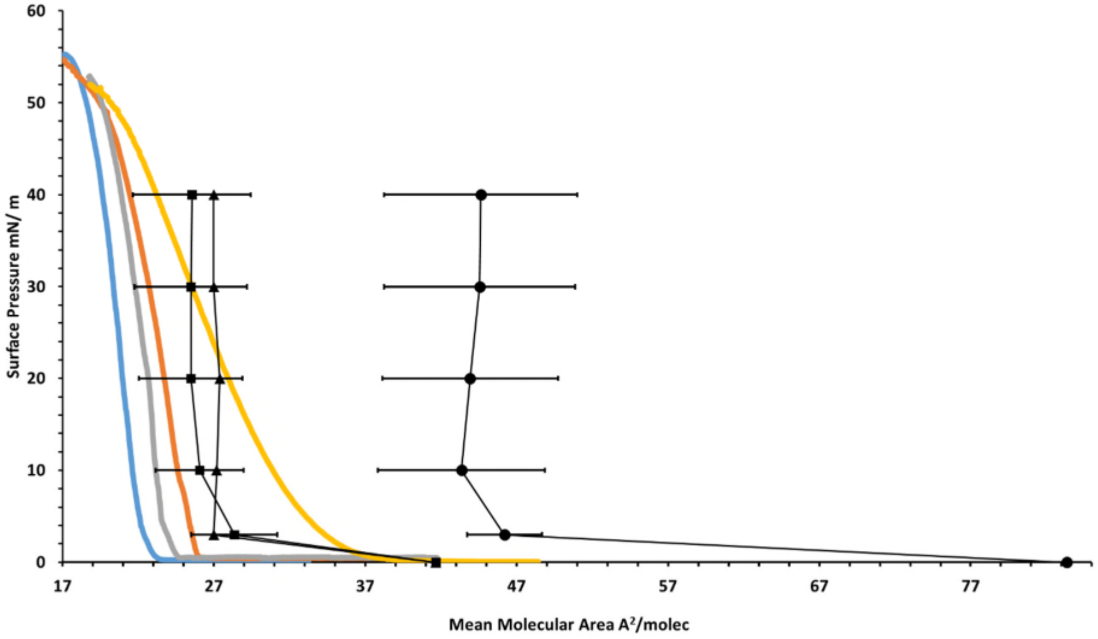

Supplementary Figure 13. Representative experimental Langmuir-Blodgett isotherms for two consecutive compression/decompression cycles on a single sample, showing surface pressure as a function of mean molecular area. Black lines are isotherms computed using MD simulations for a system containing 60 (+) rotating decacyclene molecules (■), 30 (+) rotating molecules (●), and a system with a mixture of 30 (+) and 30 (–) rotating decacyclene molecules (▲).

2.3 DFT computed geometries

Structure S1: (+)-Decacyclene-D_3
ΔE_{gas}= -1381.136632 a.u.
ΔG_{gas}= -1380.776525 a.u.

C	-1.40637100	-0.23455100	0.02338000
C	1.40114500	0.26590100	-0.02492000
C	-0.93060900	1.08032000	-0.02329000
C	-0.47023500	-1.34618900	-0.02460200
C	0.90640200	-1.10070800	0.02211200
C	0.50028500	1.33538300	0.02215400
C	0.69087500	2.79541600	0.12661000
C	-0.60253000	3.37946200	-0.00012100
C	-1.61443900	2.38453600	-0.12709000
C	-1.25811200	-2.59029900	-0.12959800
C	-2.62549100	-2.21129400	-0.00011100
C	-2.76602900	-0.79937100	0.12915300
C	2.07538900	-1.99593100	0.12741300
C	3.22808600	-1.16817800	0.00055200
C	2.87259400	0.20549700	-0.12861800
C	-0.85039800	4.77062200	-0.00024800
C	0.27579000	5.61819900	0.21631000
C	1.53238500	5.05819000	0.41971200
C	1.75972300	3.65346800	0.37631600
C	4.55681200	-1.64917900	0.00220200
C	4.72757200	-3.04794900	0.22113400
C	3.61399700	-3.85578100	0.42448300
C	2.28384400	-3.35023100	0.37920800
C	-4.04307200	-0.30310300	0.38201400
C	-5.14602600	-1.20228300	0.42561000
C	-5.00320300	-2.57019500	0.21962700
C	-3.70646700	-3.12137800	-0.00003600
C	-2.91383500	2.82040200	-0.37700000
C	-2.20005500	5.17698000	-0.21697500
C	-3.18570600	4.21721500	-0.42050600
C	-0.98657700	-3.93311200	-0.38272400
C	-3.38413400	-4.49304900	-0.21992500
C	-2.06057200	-4.86665400	-0.42650400
C	3.89993000	1.11234900	-0.37964600
C	5.58372400	-0.68385100	-0.21526700
C	5.24552500	0.64927200	-0.42149000
H	0.14399700	6.70450000	0.23456300
H	2.38495600	5.71565200	0.61406800
H	2.76398300	3.27832700	0.57510800
H	5.73422300	-3.47691400	0.24115500
H	3.75678000	-4.92256000	0.62066900
H	1.45670000	-4.03195500	0.57865500
H	-4.21986900	0.75393200	0.58256800
H	-6.14131800	-0.79291400	0.62235300
H	-5.87809900	-3.22745600	0.23827200
H	-3.72647300	2.12125000	-0.57580500

H	-2.45157100	6.24192400	-0.23552200
H	-4.21289700	4.53960000	-0.61496800
H	0.02478900	-4.28682200	-0.58430500
H	-4.18087000	-5.24307700	-0.23887300
H	-1.82675800	-5.91699800	-0.62389300
H	3.70087600	2.16525900	-0.58064400
H	6.63175900	-0.99853500	-0.23249200
H	6.03847700	1.37723800	-0.61682700

Structure S2: (–)-Decacyclene-D_3
ΔE_{gas}= -1381.136632 a.u.
ΔG_{gas}= -1380.776525 a.u.

C	-1.40637100	-0.23455100	0.02338000
C	-4.21388700	0.26590100	-0.02492000
C	-1.88213300	1.08032000	-0.02329000
C	-2.34250700	-1.34618900	-0.02460200
C	-3.71914400	-1.10070800	0.02211200
C	-3.31302700	1.33538300	0.02215400
C	-3.50361700	2.79541600	0.12661000
C	-2.21021200	3.37946200	-0.00012100
C	-1.19830300	2.38453600	-0.12709000
C	-1.55463000	-2.59029900	-0.12959800
C	-0.18725100	-2.21129400	-0.00011100
C	-0.04671300	-0.79937100	0.12915300
C	-4.88813100	-1.99593100	0.12741300
C	-6.04082800	-1.16817800	0.00055200
C	-5.68533600	0.20549700	-0.12861800
C	-1.96234400	4.77062200	-0.00024800
C	-3.08853200	5.61819900	0.21631000
C	-4.34512700	5.05819000	0.41971200
C	-4.57246500	3.65346800	0.37631600
C	-7.36955400	-1.64917900	0.00220200
C	-7.54031400	-3.04794900	0.22113400
C	-6.42673900	-3.85578100	0.42448300
C	-5.09658600	-3.35023100	0.37920800
C	1.23033000	-0.30310300	0.38201400
C	2.33328400	-1.20228300	0.42561000
C	2.19046100	-2.57019500	0.21962700
C	0.89372500	-3.12137800	-0.00003600
C	0.10109300	. 2.82040200	-0.37700000
C	-0.61268700	5.17698000	-0.21697500
C	0.37296400	4.21721500	-0.42050600
C	-1.82616500	-3.93311200	-0.38272400
C	0.57139200	-4.49304900	-0.21992500
C	-0.75217000	-4.86665400	-0.42650400
C	-6.71267200	1.11234900	-0.37964600
C	-8.39646600	-0.68385100	-0.21526700
C	-8.05826700	0.64927200	-0.42149000
H	-2.95673900	6.70450000	0.23456300
H	-5.19769800	5.71565200	0.61406800
H	-5.57672500	3.27832700	0.57510800

H	-8.54696500	-3.47691400	0.24115500
H	-6.56952200	-4.9225600	0.62066900
H	-4.26944200	-4.0319550	0.57865500
H	1.40712700	0.7539320	0.58256800
H	3.32857600	-0.7929140	0.62235300
H	3.06535700	-3.2274560	0.23827200
H	0.91373100	2.1212500	-0.57580500
H	-0.36117100	6.2419240	-0.23552200
H	1.40015500	4.5396000	-0.61496800
H	-2.83753100	-4.2868220	-0.58430500
H	1.36812800	-5.2430770	-0.23887300
H	-0.98598400	-5.9169980	-0.62389300
H	-6.51361800	2.1652590	-0.58064400
H	-9.44450100	-0.9985350	-0.23249200
H	-8.85121900	1.3772380	-0.61682700

Structure S3: Decacyclene-C_2
ΔE_{gas}= -1381.136223 a.u.
ΔG_{gas}= -1380.776133 a.u.

C	-1.42239300	-0.01001800	0.09795100
C	1.42260200	0.00160600	-0.09941400
C	-0.71100500	-1.21561400	0.12304100
C	-0.70539000	1.24994900	-0.00404900
C	0.69515800	1.25556300	0.00393900
C	0.72116400	-1.20987800	-0.12475300
C	1.14749000	-2.61105200	-0.30733500
C	0.01386400	-3.41806800	-0.00033900
C	-1.12602400	-2.61997500	0.30665200
C	-1.70521000	2.32987000	-0.11313900
C	-2.98443700	1.70695400	-0.02645700
C	-2.86787500	0.29013900	0.07700100
C	1.68594900	2.34361300	0.11393600
C	2.97030400	1.73166300	0.02626000
C	2.86554600	0.31401600	-0.07869800
C	0.01958600	-4.83079200	0.00057300
C	1.22870900	-5.46248000	-0.41558800
C	2.31633800	-4.68591900	-0.80035600
C	2.29588100	-3.26308000	-0.75091600
C	4.19274600	2.43059500	0.14947000
C	4.10682800	3.84018800	0.34661700
C	2.86087400	4.44385300	0.47286400
C	1.64457300	3.71035800	0.38095100
C	-4.04393400	-0.45482100	0.02834400
C	-5.29626200	0.21690600	-0.05130000
C	-5.39656800	1.60149700	-0.12600700
C	-4.21263800	2.39564300	-0.14962600
C	-2.26861600	-3.28065000	0.75216300
C	-1.18419600	-5.47179300	0.41796100
C	-2.27759200	-4.70358700	0.80316200
C	-1.67528100	3.69729700	-0.37868100
C	-4.13848900	3.80621200	-0.34495400

C	-2.89768100	4.42059200	-0.47003300
C	4.04791200	-0.42107600	-0.03191400
C	5.38327500	1.64640100	0.12407400
C	5.29455700	0.26113400	0.04759600
H	1.29028200	-6.55472700	-0.44452600
H	3.22844400	-5.17953100	-1.14850100
H	3.17089700	-2.71359900	-1.09986100
H	5.02192200	4.43428400	0.43247100
H	2.80494600	5.51913400	0.66645100
H	0.70550500	4.23113800	0.56832500
H	-4.03415700	-1.54428800	0.00654000
H	-6.20829800	-0.38694800	-0.07178800
H	-6.37647800	2.08331200	-0.19921100
H	-3.14760600	-2.73767700	1.10134400
H	-1.23689400	-6.56448100	0.44813900
H	-3.18535800	-5.20415300	1.15272400
H	-0.74059000	4.22621200	-0.56526400
H	-5.05855500	4.39263800	-0.43045900
H	-2.85087000	5.49652500	-0.66240800
H	4.04733900	-1.51061500	-0.01169800
H	6.35918000	2.13628400	0.19725500
H	6.21169000	-0.33498700	0.06649300

Structure S4: Decacyclene-TS
ΔE_{gas}= -1381.134731 a.u.
ΔG_{gas}= -1380.774854 a.u.
ν_i = -136.30 cm^{-1}

C	-1.41779500	-0.01217900	-0.04608400
C	1.41779300	-0.01223000	0.04607600
C	-0.71929500	1.19817400	-0.08651500
C	-0.70334500	-1.28199000	-0.00980900
C	0.70330000	-1.28201600	0.00982400
C	0.71933800	1.19814800	0.08650200
C	1.15091900	2.60102200	0.24989200
C	0.00006400	3.40343300	0.00000100
C	-1.15082000	2.60106500	-0.24990000
C	-1.72581300	-2.35607600	-0.00119500
C	-2.99796100	-1.70844400	0.01719900
C	-2.86200200	-0.29263300	0.02310900
C	1.72573300	-2.35613600	0.00122100
C	2.99790200	-1.70854500	-0.01719500
C	2.86198900	-0.29273100	-0.02312700
C	0.00008500	4.81638600	0.00001500
C	1.22376600	5.45265900	0.36281900
C	2.33023800	4.68004200	0.69845900
C	2.31458600	3.25724400	0.64526000
C	4.24034100	-2.38014600	-0.08869400
C	4.20054200	-3.80425100	-0.08497600
C	2.97316000	-4.45312600	-0.04717700
C	1.73695300	-3.74925900	-0.01796700
C	-4.01759100	0.46867500	0.18481800

C	-5.27999600	-0.18324300	0.25625400
C	-5.40631900	-1.56590500	0.19105500
C	-4.24042400	-2.38000300	0.08868700
C	-2.31446400	3.25733200	-0.64525800
C	-1.22357700	5.45270600	-0.36277200
C	-2.33007400	4.68013100	-0.69842900
C	-1.73708200	-3.74919900	0.01801100
C	-4.20067500	-3.80410800	0.08499200
C	-2.97331400	-4.45302500	0.04721900
C	4.01760000	0.46853600	-0.18488000
C	5.40626100	-1.56609100	-0.19110200
C	5.27998200	-0.18342600	-0.25632900
H	1.28187900	6.54513400	0.39065300
H	3.25453800	5.17683700	1.00777300
H	3.20741300	2.71092900	0.95136000
H	5.13231100	-4.37682000	-0.12464700
H	2.94426300	-5.54669000	-0.05325800
H	0.82402300	-4.33940300	-0.01960700
H	-3.97991700	1.55198100	0.29817000
H	-6.17719800	0.43184900	0.37236000
H	-6.39311600	-2.03619600	0.24370900
H	-3.20730600	2.71105000	-0.95137400
H	-1.28165500	6.54518300	-0.39058100
H	-3.25435800	5.17696100	-1.00773200
H	-0.82417700	-4.33938300	0.01966500
H	-5.13246400	-4.37664600	0.12465700
H	-2.94445200	-5.54658900	0.05331500
H	3.97995700	1.55184200	-0.29825900
H	6.39304100	-2.03641600	-0.24376800
H	6.17720100	0.43163400	-0.37247100

Structure S5: Decacyclene_dimer
ΔE_{gas}= -2762.326065 a.u.
ΔG_{gas}= -2761.577693 a.u.

C	4.90657400	-3.95731700	-0.64775500
C	2.58933500	-2.38209500	-1.19622900
C	5.07038500	-2.55737300	-0.86509800
C	3.63177300	-4.51165800	-0.66155100
C	2.46433700	-3.74356500	-0.93404400
C	3.89277300	-1.81362800	-1.10061900
C	3.85622100	-0.39804800	-1.25144800
C	5.07552300	0.27474900	-1.28889400
C	6.28135400	-0.45517800	-1.09167600
C	6.29466400	-1.82663300	-0.85929800
C	2.43366400	-0.03215400	-1.39419700
C	1.66774800	-1.26342200	-1.47706200
C	1.77539700	1.20020000	-1.43926700
C	0.34909800	1.25566700	-1.72226600
C	-0.38539100	0.06899600	-1.82980500
C	0.30004700	-1.21321300	-1.76205300
C	-0.68869700	-2.26779700	-2.05289300

C	-1.95118100	-1.61728800	-2.15871100
C	-1.82683400	-0.20690100	-1.99557400
C	-0.02363500	2.67998800	-1.84444100
C	1.13767900	3.43148300	-1.50168600
C	2.24413400	2.58059600	-1.21658100
C	-3.17362200	-2.28892800	-2.38471200
C	-3.09649400	-3.69660200	-2.59439200
C	-1.85946200	-4.33274700	-2.57533600
C	-0.64810300	-3.63832800	-2.30105000
C	1.18665500	4.84208200	-1.44645400
C	0.00262500	5.53525200	-1.83232900
C	-1.11650700	4.81643900	-2.23653600
C	-1.14976200	3.39379200	-2.24685300
C	-4.35271700	-1.48742700	-2.37167400
C	-3.00389700	0.53636500	-1.95421300
C	-4.25318000	-0.12050200	-2.13998500
C	3.41184100	3.18079300	-0.74892200
C	2.41226400	5.42087300	-1.00393200
C	3.47570800	4.59937600	-0.64703300
C	3.10295500	-3.68932000	2.59512600
C	0.69267300	-2.26480900	2.05342500
C	3.17764800	-2.28156200	2.38511600
C	1.86702000	-4.32759900	2.57632000
C	0.65444500	-3.63534100	2.30195300
C	1.95402800	-1.61209400	2.15901100
C	1.82722400	-0.20195400	1.99565200
C	3.00298400	0.54338000	1.95428100
C	4.25340900	-0.11127900	2.14008800
C	4.35533700	-1.47800900	2.37191900
C	0.38530200	0.07141000	1.82980800
C	-0.29791000	-1.21200600	1.76237800
C	-1.66552300	-1.26468200	1.47742300
C	-2.43356400	-0.03477000	1.39418200
C	-1.77743400	1.19873700	1.43885400
C	-0.35125100	1.25677800	1.72191500
C	0.01898300	2.68180000	1.84363100
C	-1.14361400	3.43114500	1.50051100
C	-2.24856000	2.57823000	1.21562800
C	-3.85549400	-0.40314500	1.25162500
C	-3.88961800	-1.81884400	1.10129700
C	-2.58519400	-2.38502800	1.19698500
C	-2.45783600	-3.74635900	0.93521100
C	-3.62395400	-4.51657100	0.66305800
C	-4.89971600	-3.96444800	0.64921300
C	-5.06594300	-2.56471700	0.86610400
C	-1.19513000	4.84164100	1.44494000
C	-0.01239400	5.53703500	1.83079600
C	1.10796100	4.82032100	2.23533900
C	1.14375500	3.39773600	2.24604100
C	-3.41732900	3.17621200	0.74778300
C	-2.42174700	5.41812900	1.00221400
C	-3.48371700	4.59464700	0.64549100

C	-5.07596600	0.26754100	1.28890100
C	-6.29149900	-1.83611000	0.86013100
C	-6.28054500	-0.46455300	1.09202100
H	3.51119200	-5.57914100	-0.45482100
H	1.49122700	-4.23110800	-0.87802800
H	5.13593300	1.34328300	-1.49712800
H	7.22905300	0.09109700	-1.11917500
H	7.24056200	-2.35057400	-0.68919000
H	-4.01139300	-4.26897800	-2.77402600
H	-1.80947400	-5.40923200	-2.76499700
H	0.29206900	-4.18987300	-2.32656200
H	-0.01894800	6.62927400	-1.81179000
H	-2.01657400	5.35667600	-2.54326800
H	-2.05229300	2.89422300	-2.59756500
H	-5.33113300	-1.95511900	-2.51507200
H	-3.00205600	1.60178700	-1.72456100
H	-5.16668100	0.47834500	-2.08647600
H	4.27206600	2.59128000	-0.43035200
H	4.40240800	5.05243200	-0.28193200
H	1.81891900	-5.40412400	2.76624300
H	-0.28478600	-4.18847800	2.32768300
H	2.99919000	1.60880200	1.72468300
H	5.16587300	0.48914600	2.08654100
H	5.33457500	-1.94397200	2.51533200
H	-1.48387200	-4.23220100	0.87923500
H	-3.50152600	-5.58390000	0.45663500
H	-5.77424200	-4.59323600	0.45448800
H	0.00722500	6.63108900	1.80998800
H	2.00701800	5.36223500	2.54208200
H	2.04711500	2.89990200	2.59709200
H	-4.27650500	2.58507200	0.42939900
H	-2.51543800	6.50614200	0.92925200
H	-4.41121300	5.04595600	0.28025100
H	-5.13825300	1.33604400	1.49675700
H	-7.23649500	-2.36175100	0.69025700
H	-7.22917800	0.08010700	1.11939800
H	5.78216200	-4.58454000	-0.45276100
H	2.50402000	6.50906700	-0.93123600
H	4.01884800	-4.26008600	2.77481400

Supplementary References

1. Amick AW, Scott LT. Trisannulated benzene derivatives by acid catalyzed aldol cyclotrimerizations of cyclic ketones. Methodology development and mechanistic insight. *J Org Chem* 2007, **72**(9)**:** 3412-3418.

2. Hill TJ, Hughes RK, Scott LT. Steps toward the synthesis of a geodesic C60H12 end cap for a C3v carbon [6, 6] nanotube. *Tetrahedron* 2008, **64**(50)**:** 11360-11369.

3. Pantelic RS, Suk JW, Magnuson CW, Meyer JC, Wachsmuth P, Kaiser U*, et al.* Graphene: substrate preparation and introduction. *J Struct Biol* 2011, **174**(1)**:** 234-238.

4. Lee C, Wei X, Kysar JW, Hone J. Measurement of the elastic properties and intrinsic strength of monolayer graphene. *science* 2008, **321**(5887)**:** 385-388.

5. Roos WH, Bruinsma R, Wuite GJL. Physical virology. *Nature physics* 2010, **6**(10)**:** 733-743.

6. Roos WH. How to perform a nanoindentation experiment on a virus. *Single Molecule Analysis*. Springer, **2011**, pp 251-264.

7. Frisch MJ, Trucks GW, Schlegel HB, Scuseria GE, Robb MA, Cheeseman JR*, et al.* Gaussian 09 Revision D. 01, 2009. *Gaussian Inc Wallingford CT* 2009.

8. Grimme S, Antony J, Ehrlich S, Krieg H. A consistent and accurate ab initio parametrization of density functional dispersion correction (DFT-D) for the 94 elements H-Pu. *J Chem Phys* 2010, **132**(15)**:** 154104.

9. Grimme S, Ehrlich S, Goerigk L. Effect of the damping function in dispersion corrected density functional theory. *J Comp Chem* 2011, **32**(7)**:** 1456-1465.

10. Ribeiro RF, Marenich AV, Cramer CJ, Truhlar DG. Use of solution-phase vibrational frequencies in continuum models for the free energy of solvation. *J Phys Chem B* 2011, **115**(49)**:** 14556-14562.

11. Funes-Ardoiz, I.; Paton, R. S., **2016**. GoodVibes: GoodVibes v1.0.2. http://doi.org/10.5281/zenodo.595246.

12. Legault CY. *CYLview, 1.0 b, Université de Sherbrooke, 2009*, 2013.

13. Berendsen HJC, van der Spoel D, van Drunen R. GROMACS: a message-passing parallel molecular dynamics implementation. *Comput Phys Commun* 1995, **91**(1-3)**:** 43-56.

14. Lindahl E, Hess B, van Der Spoel D. GROMACS 3.0: a package for molecular simulation and trajectory analysis. *Mol Mod Ann* 2001, **7**(8)**:** 306-317.

15. van Der Spoel D, Lindahl E, Hess B, Groenhof G, Mark AE, Berendsen HJC. GROMACS: fast, flexible, and free. *J Comp Chem* 2005, **26**(16)**:** 1701-1718.

16. Hess B, Kutzner C, van Der Spoel D, Lindahl E. GROMACS 4: algorithms for highly efficient, load-balanced, and scalable molecular simulation. *J Chem Theo Comp* 2008, **4**(3)**:** 435-447.

17. Pronk S, Páll S, Schulz R, Larsson P, Bjelkmar P, Apostolov R*, et al.* GROMACS 4.5: a high-throughput and highly parallel open source molecular simulation toolkit. *Bioinformatics* 2013, **29**(7)**:** 845-854.

18. Abraham MJ, Murtola T, Schulz R, Páll S, Smith JC, Hess B*, et al.* GROMACS: High performance molecular simulations through multi-level parallelism from laptops to supercomputers. *SoftwareX* 2015, **1:** 19-25.

19. Páll S, Abraham MJ, Kutzner C, Hess B, Lindahl E. Tackling exascale software challenges in molecular dynamics simulations with GROMACS. 2014: Springer. p. 3-27.

20. York DM, Darden TA, Pedersen LG. The effect of long-range electrostatic interactions in simulations of macromolecular crystals: A comparison of the Ewald and truncated list methods. *J Chem Phys* 1993, **99**(10)**:** 8345-8348.

21. Bussi G, Donadio D, Parrinello M. Canonical sampling through velocity rescaling. *J Chem Phys* 2007, **126**(1)**:** 014101.

22. Horn HW, Swope WC, Pitera JW, Madura JD, Dick TJ, Hura GL*, et al.* Development of an improved four-site water model for biomolecular simulations: TIP4P-Ew. *J Chem Phys* 2004, **120**(20)**:** 9665-9678.

23. Harris JG. Liquid-vapor interfaces of alkane oligomers: structure and thermodynamics from molecular dynamics simulations of chemically realistic models. *J Phys Chem* 1992, **96**(12)**:** 5077-5086.

24. Vega C, De Miguel E. Surface tension of the most popular models of water by using the test-area simulation method. *J Chem Phys* 2007, **126**(15)**:** 154707.

25. Chen F, Smith PE. Simulated surface tensions of common water models. *J Chem Phys* 2007, **126:** 221101.

26. Jorgensen WL, Maxwell DS, Tirado-Rives J. Development and testing of the OPLS all-atom force field on conformational energetics and properties of organic liquids. *J Am Chem Soc* 1996, **118**(45)**:** 11225-11236.

27. Kaminski GA, Friesner RA, Tirado-Rives J, Jorgensen WL. Evaluation and reparametrization of the OPLS-AA force field for proteins via comparison with accurate quantum chemical calculations on peptides. *J Phys Chem B* 2001, **105**(28)**:** 6474-6487.

28. Martin MG. Comparison of the AMBER, CHARMM, COMPASS, GROMOS, OPLS, TraPPE and UFF force fields for prediction of vapor–liquid coexistence curves and liquid densities. *Fluid Phase Equilibr* 2006, **248**(1)**:** 50-55.

29. Dodda LS, Cabeza de Vaca I, Tirado-Rives J, Jorgensen WL. LigParGen web server: an automatic OPLS-AA parameter generator for organic ligands. *Nucleic Acid Res* 2017, **45**(W1)**:** W331-W336.

30. Marenich AV, Jerome SV, Cramer CJ, Truhlar DG. Charge model 5: An extension of Hirshfeld population analysis for the accurate description of molecular interactions in gaseous and condensed phases. *J Chem Theo Comp* 2012, **8**(2)**:** 527-541.

31. Frisch MJ, Trucks GW, Schlegel HB, Scuseria GE, Robb MA, Cheeseman JR*, et al.* Gaussian 16, revision C. 01. Gaussian, Inc., Wallingford CT; 2016.

32. Martínez JM, Martínez L. Packing optimization for automated generation of complex system's initial configurations for molecular dynamics and docking. *J Comp Chem* 2003, **24**(7)**:** 819-825.

33. Martínez L, Andrade R, Birgin EG, Martínez JM. PACKMOL: a package for building initial configurations for molecular dynamics simulations. *J Comp Chem* 2009, **30**(13)**:** 2157-2164.

34. van der Spoel D, Lindahl E, Hess B, van Buuren AR, Apol E, Meulenhoff PJ*, et al. Gromacs User Manual version 4.5. 6, 2010*.

35. Fine RA, Millero FJ. Compressibility of water as a function of temperature and pressure. *J Chem Phys* 1973, **59**(10)**:** 5529-5536.

36. Humphrey W, Dalke A, Schulten K. VMD: visual molecular dynamics. *J Mol Graphics* 1996, **14**(1)**:** 33-38.

37. Gowers RJ, Linke M, Barnoud J, Reddy TJE, Melo MN, Seyler SL*, et al. MDAnalysis: a Python package for the rapid analysis of molecular dynamics simulations*. Los Alamos National Lab.(LANL), Los Alamos, NM (United States), 2019.

38. Ribeiro AAST, Horta BAC, Alencastro RBd. MKTOP: a program for automatic construction of molecular topologies. *J Brazil Chem Soc* 2008, **19**(7)**:** 1433-1435.

39. Jorgensen WL, Severance DL. Aromatic-aromatic interactions: free energy profiles for the benzene dimer in water, chloroform, and liquid benzene. *J Am Chem Soc* 1990, **112**(12)**:** 4768-4774.
